# Is quality of life different between diabetic and non-diabetic people? The importance of cardiovascular risks

**DOI:** 10.1371/journal.pone.0189505

**Published:** 2017-12-14

**Authors:** L. M. Peña-Longobardo, B. Rodríguez-Sánchez, M. Mata-Cases, L. Rodríguez-Mañas, M. Capel, J. Oliva-Moreno

**Affiliations:** 1 University of Castilla-La Mancha, Department of Economic Analysis and Finance, Toledo, Spain; 2 University of Groningen, Department of Economics, Econometrics and Finance, Groningen, The Netherlands; 3 Primary Health Care Center La Mina, Gerència d’Àmbit d’Atenció Primària Barcelona Ciutat, Institut Català de la Salut, Barcelona, Spain; 4 DAP-Cat Group, Unitat de Suport a la Recerca Barcelona Ciutat, Institut Universitari d’Investigació en Atenció Primària Jordi Gol (IDIAP Jordi Gol), Barcelona, Spain; 5 CIBER of Diabetes and Associated Metabolic Diseases (CIBERDEM), Instituto de Salud Carlos III (ISCIII), Madrid, Spain; 6 Geriatrics Service, Getafe University Hospital, Madrid, Spain; 7 Astrazeneca, Health Economics and Outcomes Research, Madrid, Spain; Universitat de Valencia, SPAIN

## Abstract

**Background:**

To analyse and compare the impact of cardiovascular risk factors and disease on health-related quality of life (HRQoL) in people with and without diabetes living in the community.

**Methods:**

We used data of 1,905 people with diabetes and 19,031 people without diabetes from the last Spanish National Health Survey (years 2011–2012). The HRQoL instrument used was the EuroQol 5D-5L, based on time trade-off scores. Matching methods were used to assess any differences in the HRQoL in people with and without diabetes with the same characteristics (age, gender, education level, and healthy lifestyle), according to cardiovascular risk factors and diseases. Disparities were also analysed for every dimension of HRQoL: mobility, daily activities, personal care, pain/discomfort, and anxiety/depression.

**Results:**

There were no significant differences in time trade-off scores between people with and without diabetes when cardiovascular risk factors or established cardiovascular disease were not present. However, when cardiovascular risk factors were present, the HRQoL score was significantly lower in people with diabetes than in those without. This difference was indeed greater when cardiovascular diseases were present. More precisely, people with diabetes and any of the cardiovascular risk factors, who have not yet developed any cardiovascular disease, report lower HRQoL, 0.046 TTO score points over 1 (7.93 over 100 in the VAS score) compared to those without diabetes, and 0.14 TTO score points of difference (14.61 over 100 in the VAS score) if cardiovascular diseases were present. In fact, when the three risk factors were present in people with diabetes, HRQoL was significantly lower (0.10 TTO score points over 1 and 10.86 points over 100 in VAS score), obesity being the most influential risk factor.

**Conclusions:**

The presence of established cardiovascular disease and/or cardiovascular risk factors, specially obesity, account for impaired quality of life in people with diabetes.

## Introduction

Diabetes Mellitus leads to complications associated with high social costs which greatly affect individual and social wellbeing [1–9], reducing quality of life (QoL). Diabetes might lead to eyesight loss due to retinopathy, the need for dialysis because of kidney failure, or amputation because of peripheral vascular disease [6, 10]. In addition, more than 50% of patients with type 2 diabetes are over 65 years of age–as high as 62% in a recent study conducted in Catalonia that used a population database [11]. Thus, functional impairment is a major problem in diabetes affecting individual autonomy and QoL [11, 12], disease-associated costs [13,14], and with a significant prognostic value [14].

Given the complications related to diabetes and their consequences in decreasing autonomy and wellbeing, analysing how diabetes affects health-related QoL (HRQoL) should be of great interest not only to those with the disease, but also to health policymakers and society as a whole. In recent years, the number of studies focused on measuring and assessing HRQoL in people with diabetes has grown [10, 15]. The studies concluded that QoL is negatively affected by diabetes, regardless of the evaluation tool, and that the combined prevalence of diabetes and cardiovascular diseases is associated with a decrease in HRQoL [16, 17].

However, despite the increase in the number of studies analysing the negative association between HRQoL and diabetes in the last decade, the literature comparing the HRQoL of people with and without diabetes remains very scarce as well as the analyses of the potential factors involved in this negative effect, usually limited to the role of cardiovascular disease, but ignoring the one due to cardiovascular risk factors [16, 18, 19]. Moreover, no studies have compared the HRQoL in people with and without diabetes using a representative sample of the general population, with the use of advanced statistical methods appropriate for this purpose and identifying the dimension of HRQoL (mobility, self-care, daily activities, pain/discomfort, or anxiety/depression) which are affected the most. Therefore, the main objective of this study is to address these shortcomings by analysing and comparing the impact of both cardiovascular risk factors and diseases on HRQoL and its domains of people with and without diabetes.

## Methods

### Data

Data from the last Spanish National Health Survey conducted by the National Statistics Institute in Spain was used [20]. The sample included 21,508 households with 26,502 interviews; there were 21,007 adults (15 years or older) and 5,495 children (0–14 years). The recruitment took place from July 2011 to June 2012 and comprised questionnaires for the households, adults, and children. This is a cross-sectional survey, representative at a national level, and provides information on sociodemographic characteristics, physical and mental health, variables related to lifestyle (smoking, alcohol consumption, exercise), chronic diseases, and use of healthcare resources. Due to it is a National Public sample, it was not necessary to seek an ethics committee.

The variables of interest of this study are based on the EuroQol (EQ)-5D-5L, which is a simple generic instrument developed by a multidisciplinary group of researchers. HRQoL is defined in terms of 5 dimensions: mobility, self-care, daily activities, pain/discomfort, and anxiety/depression. The values and utilities are assigned a score on a scale based on responders’ self-reported health, where 0 corresponds to death and 1 corresponds to perfect health, with negative values being possible. The second part of the EQ-5D-5L consists of a visual analogue scale (VAS), on which 0 represents the worst and 100 represents the best imaginable health state. Respondents mark a point on the scale to reflect their overall perception of health on the day of the interview [21].

In this study, people with diabetes are defined as those who answered affirmatively to either one of the following two questions: "Have you ever suffered from diabetes?" or "Have you ever taken medicines for diabetes?" To define cardiovascular diseases, we include those individuals who answered affirmatively to any of the following questions: "have you ever had (i) myocardial infarction; (ii) other heart diseases; or (iii) embolism, stroke or brain haemorrhage?" Similarly, individuals with cardiovascular risk factors are identified by the following questions: "Have you ever had (i) high cholesterol or (ii) hypertension?”; (iii) a body mass index (BMI) of 30 kg/m^2^ or more is also considered a risk factor.

In the descriptive analysis, since the average age is much higher in people with diabetes, based on the age distribution of people with diabetes observed in the sample (on average, 68), S1 Table (supplementary material), a third group of people of 55 years and older without diabetes has also been defined in order to compare two different groups with similar age.

### Statistical analysis

Different propensity matching score regressions have been applied to analyse the differences in the HRQoL and VAS scores between people with and without diabetes. The different comparison groups are shown in Table 1. Due to the case assignment (being diagnosed with diabetes), this is not a random process, and the differences between individuals in the treatment and control groups have been recorded with observable variables (such as gender, age, education, and healthy lifestyle); a matching technique is therefore the most suitable method [22–24].

**Table 1:** Prevalence of cardiovascular risk factors or disease in three defined groups of people with and without diabetes.

Groups	Prevalence by subgroup (%)
People with diabetes and without cardiovascular risk factors or disease	11.9%
People with diabetes with cardiovascular risk factors and without cardiovascular disease	63.6%
People with diabetes and established cardiovascular disease	24.5%
People without diabetes without cardiovascular risk factors or cardiovascular disease	54.6%
People without diabetes with cardiovascular risk factors and without cardiovascular disease	36.9%
People without diabetes with established cardiovascular disease	8.5%
People without diabetes aged ≥55 without cardiovascular risk factors or established cardiovascular disease	27.0%
People without diabetes aged ≥55 with cardiovascular risk factors and without established cardiovascular disease	55.1%
People without diabetes aged ≥55 with established cardiovascular disease	17.9%

Source: Authors’ version, based on the 2012 National Health Survey

Since we study both treated (different comparison groups) and untreated individuals (control group: not diagnosed with diabetes and no cardiovascular risk factors or disease), we are unable to note a causal effect of interest: Y_i1_-Y_i0_, with Y_i1_ being the effect on the comparison group and Y_i0_ being the effect on the control group. We may thus consider the Average Treatment Effect *on the Treatment Group* (ATE):*E*(*Y*_1_ − *Y*_0_|*X*, *D* = 1) = *E*(Δ|*X*, *D* = 1), where D is a dummy variable indicating status as belonging to the comparison group (1) or belonging to the control group (0), and X is a vector of observable characteristics. This equation measures the difference in the average outcome (HRQoL) of people in the comparison and control groups.

The matching method could have been difficult to accomplish if determined by multiple variables (meaning matching all participants in the treatment group with all participants in the control group with the same characteristics). To avoid this problem of dimensionality, it has been necessary to use the propensity score for determination purposes, demonstrating that if (*Y*_0_, *Y*_1_) ⊥ *D*|*X* and 0 <P (X) <1, where P(*X*) = Pr(*D* = 1|*X*), then, (*Y*_0_, *Y*_1_) ⊥ *D*|P(*X*); i.e., the result for HRQoL is the same both for individuals belonging to the different comparison groups and those belonging to the control group after adjusting for the observable characteristics (X variables) [25].

The nearest-neighbour matching technique is used, as it has lower pseudo-R squared in the probit estimation for propensity score after matching, and a lower number of *treated* individuals lost after matching.

## Results

Out of the 20,936 people who had completed the EQ-5D-5L questionnaire appropriately, 1,905 (9.1% of the valid responses) had diabetes and 19,031 do not have diabetes. The main sociodemographic characteristics of the sample are described in S1 Table (supporting information).

The prevalence of any risk factors or cardiovascular diseases among these three groups show remarkable differences (Table 1). While only 11.9% of the population with diabetes did not have any cardiovascular risk factors or disease, 63.6% had any of the cardiovascular risk factors but free from cardiovascular events and 24.5% had ever had a previous cardiovascular episode. In contrast, 54.6% of those without diabetes had no cardiovascular risk factor nor cardiovascular disease, 36.9% suffer from any cardiovascular risk factor, and only 8.5% had ever had a previous cardiovascular episode. Finally, among people without diabetes aged over 55 years, 27.0% had no cardiovascular risk factors nor cardiovascular disease, 55.1% had cardiovascular risk factors, and 17.9% had ever suffered from a cardiovascular disease.

Table 2 shows the raw average score of the EQ-5D-5L in the three groups considered. People with diabetes achieve 0.781 total points in the time trade-off (TTO) score and 61 on the VAS. Individuals without diabetes achieve higher results in both the TTO (0.921) and VAS scores (77). Similarly, people without diabetes of 55 years and older had higher scores than people with diabetes, but the scores were lower than those for the total sample of people without diabetes (0.858 and 69 for TTO and VAS scores, respectively).

**Table 2:** Assessment of health-related quality of life (EuroQol-5D-5L score) in three defined groups of people with and without diabetes.

	People with DM Mean (SD)	People without DM Mean (SD)	People without DM ≥55 years old) Mean (SD)
**EQ-5D-5L-TTO Score**	0.781 (0.247)	0.921 (0.156)	0.858 (0.201)
**VAS (from 0 to 100)**	61.24 (20.43)	77.17 (18.17)	69.039 (19.325)
** Mobility (%)**			
No problems in walking about	53.7	85.4	69.5
Have slight problems in walking about	16.6	6.9	14.1
Have moderate problems in walking about	15.7	4.6	10.0
Have severe problems in walking about	10.9	2.3	5.0
Unable to walk about	2.94	0.6	1.2
** Self-Care (%)**			
No problems washing or dressing myself	76.9	93.7	86.2
Have slight problems washing or dressing myself	8.7	2.9	6.5
Have moderate problems washing or dressing myself	6.6	1.6	3.7
Have severe problems washing or dressing myself	4.5	0.8	1.8
Unable to wash or dress myself	3.2	0.7	1.7
** Usual activities (%)**			
No problems doing my usual activities	63.3	88.7	77.3
Have slight problems doing my usual activities	14.6	5.3	10.6
Have moderate problems doing my usual activities	10.8	3.1	6.4
Have severe problems doing my usual activities	5.3	1.4	2.9
Unable to do my usual activities	5.8	1.1	2.5
** Pain/Discomfort (%)**			
No pain or discomfort	45.7	74.4	58.7
Have slight pain or discomfort	22.6	13.1	19.5
Have moderate pain or discomfort	20.1	8.8	14.8
Have severe pain or discomfort	10.1	3.2	6.2
Have extreme pain or discomfort	1.3	0.3	0.6
** Anxiety/Depression (%)**			
Not anxious or depressed	71.0	85.0	78.4
Slightly anxious or depressed	14.7	8.7	11.9
Moderately anxious or depressed	9.7	4.2	6.4
Severely anxious or depressed	3.8	1.6	2.4
Extremely anxious or depressed	0.7	0.4	0.7

Source: Authors’ version, based on the National Health Survey

When we assess the domains of the EQ-5D-5L, several differences between the three groups of individuals have been noted (Table 2).

Table 3 shows the results obtained after the application of statistical matching methods, displaying differences in TTO and VAS scores of patients with diabetes and the control groups, considering the presence or absence of cardiovascular risk factors or disease. The results showed statistically significant differences in the TTO scores between people with diabetes and people in the control groups. More precisely, individuals with diabetes scored on average 0.069 points less on the scale of TTO scores, compared to people without diabetes or any cardiovascular risk factors (7.94 lower in the VAS scale).

**Table 3:** Results from matching regression analysis according to time trade-off scores and VAS responses on the EuroQol-5D-5L.

	TTO score	VAS score
Comparison groups	Marginal effect (SE)	Marginal effect (SE)
People with diabetes vs. control group^a^	-0.0689 (0.007) ^b^	-7.935 (0.6774) ^b^
People with diabetes without cardiovascular risk factors or established cardiovascular disease vs. control group	0.0144 (0.017)	-2.090 (1.922)
People with diabetes with cardiovascular risk factors and without cardiovascular events vs. control group	-0.0459 (0.009) ^b^	-5.9899 (0.8441) ^b^
People with diabetes with cardiovascular events vs. control group	-0.1412 (0.016) ^b^	-14.6191 (1.277) ^b^
People with diabetes with 1 cardiovascular risk factor vs. control group	-0.0145 (0.013) ^b^	-4.4707 (1.250) ^b^
People with diabetes with 2 cardiovascular risk factors vs. control group	-0.0513 (0.014) ^b^	-6.529 (1.346) ^b^
People with diabetes with 3 cardiovascular risk factors vs. control group	-0.1005 (0.0242) ^b^	-10.8647 (2.2125) ^b^
People without diabetes with cardiovascular risk factors and without cardiovascular events vs. the control group	-0.0165 (0.002) ^b^	-2.9871 (0.3322) ^b^
People without diabetes with cardiovascular events vs. control group	-0.1221 (0.024) ^b^	-9.8157 (2.093) ^b^

^a^ people without diabetes with neither cardiovascular risk factor nor cardiovascular events

^b^Statistically significant at 99%. Source: Authors’ version, based on the National Health Survey Note: The values of TTO goes from 0 (death) and 1 (the best health status), with negative values being possible, indicating health states worse than death. The values of VAS ranges from, 0 represents the worst and 100 represents the best imaginable health state.

There were no differences in HRQoL between people with diabetes and without cardiovascular risk factors or cardiovascular events and the control group. When people with diabetes had any of the risk factors, their HRQoL was 0.046 points lower than that of the control group (5.99 lower in the VAS scale) and when people with diabetes have any cardiovascular disease their HRQoL was 0.1412 points lower than that of the control group (14.62 lower in the VAS scale).

We also analysed the cumulative effect of the presence of one, two, or three of the cardiovascular risk factors considered (obesity, hypertension, and/or cholesterol). We had found a dose-response relationship; people with diabetes and only one cardiovascular risk factor had, on average, 0.014 points less on the HRQoL scale compared to the control group (4.47 lower in the VAS scale), reaching a difference of 0.051 points when two cardiovascular risk factors were present (6.52 lower in the VAS scale) and 0.105 if all the three considered factors were reported (10.86 lower in the VAS scale). Of the 0.046 less points in the HRQoL that people with diabetes, with cardiovascular risk factors and without cardiovascular events have, compared to the control group, obesity was the most influential risk factor in the deterioration of HRQoL (see S1 Table in supporting information). More precisely, the presence of diabetes plus obesity reduced the HRQoL by 0.042 compared to the control group. The presence of diabetes, obesity and hypertension reduce the HRQoL by 0.06 points compared to the control group.

In general, the results of such a poorer quality of life in people with diabetes should be interpreted taking into consideration the results obtained in Table 1, that is, people with DM have more cardiovascular risks and events compared to the general population (Tables 1 & 3).

An analysis of differences in each dimension of HRQOL (mobility, self-care, conducting daily activities, pain/discomfort, and feeling depressed and/or anxious) has also been performed (see supporting information S3–S7 Tables). The main dimensions accounting for the differences in HRQOL were mobility, carrying out usual activities, and pain and/or discomfort. People with diabetes were 14.5 percentage points less likely to have no movement problem compared to people without diabetes or cardiovascular risk factors. Similarly, differences were also observed in having no problems in performing usual activities (11.2 percentage points lower in people with diabetes) and pain and/or discomfort (15.0 percentage points lower in people with diabetes).

## Discussion

Generally, in addition to confirm the deleterious effect of diabetes on quality of life and the role of established cardiovascular disease, our results add several novel factors: (i) a comparison of HRQoL in people with and without diabetes, using a representative sample of the general population in Spain; (ii) use of advanced statistical methods for this specific purpose; (iii) identification of the relevance of cardiovascular risk factors in people with diabetes; and (iv) identification of the dimensions of HRQoL affected by diabetes as compared with people without the disease.

The results of our study confirm that HRQoL is significantly poorer in people with diabetes. Moreover, the presence of cardiovascular risk factors or diseases leads to significantly reduced HRQoL in people with diabetes, in comparison to their counterparts. When people with diabetes do not have any risk factors or cardiovascular disease, there is no difference in terms of HRQoL between people with and without diabetes. However, people with diabetes and any of the cardiovascular risk factors, who have not yet developed any cardiovascular disease, report lower HRQoL (0.046 points) compared to the control group. In fact, when the three risk factors are present in people with diabetes, the HRQoL is significantly lower (0.10 points). Among the risks factors, obesity is the most influential one in the deterioration of HRQoL. More precisely, the presence of diabetes plus obesity reduces the HRQoL by 0.042 compared to the control group. When cardiovascular diseases are present, the difference in the TTO score reaches the highest level, 0.141 lower than the control group. These results are in line with the literature [26].

In brief, our results are consistent with those in the literature about QoL and cardiovascular disease in people with diabetes; signalling a greater decrease in HRQoL in individuals with diabetes who also have cardiovascular diseases [10, 15, 27–30], showing the role of cardiovascular risk factors in determining lower QoL. Such poorer quality of life in people with diabetes can mainly be explained by the fact that people with DM have more cardiovascular risks and events compared to the general population.

Our results are also consistent with previous studies in which a negative association between HRQoL and the type of cardiovascular disease in people with diabetes has already been reported. The determinants of lower HRQoL in people with diabetes include both microvascular (retinopathy, neuropathy, and nephropathy) and macrovascular diseases (coronary heart disease, cerebrovascular disease, and peripheral artery disease) [27]. More specifically, HRQoL in people with diabetes and congestive heart disease is reduced by 0.185 when the latter is the only comorbidity, and by 0.297 if there is an additional disease [31]. A Norwegian report has showed that the factors that reduce QoL the most are stroke and neuropathy for both diabetes types, and ischaemic heart disease for type 1 diabetes [32]. The association between cardiovascular diseases, diabetes, and QoL has also been shown in the KORA study of a German population, in which stroke, neuropathy, and myocardial infarction more negatively affect the QoL of individuals with diabetes [28]. Similarly, British researchers from the United Kingdom Prospective Diabetes Study (UKPDS) have reported the same negative impact of cardiovascular diseases [29].

It is hard to compare the results of this study with previous studies due to the lack of literature comparing HRQOL in populations with or without diabetes. The only previous Spanish study evaluating HRQoL in people with and without diabetes was conducted in 2003 [30]. In the CODE-2 Spanish, 1,000 participants cared for in primary care centres completed the EQ-5D-5L. When comparing people with type 2 diabetes with a matched population without diabetes, the former had worse scores in all components of the EQ-5D-5L; the scores were especially poor in those with diabetes and chronic micro- and macrovascular complications.

According to the data used in our analysis, other chronic diseases such as osteoarthritis, COPD or tumours have been found to also reduce HRQoL in 0.179, 0.1414 and 0.1312 TTO score points, respectively. These results are in line with literature. Those suffering from COPD have between 0.05 and 0.18 (depending on the severity of the disease) TTO score points less compared to those who do not [33, 34]. Alike, those with osteoarthritis have on average 0.08 TTO [35]. Apparently, it may seem that the loss of HRQoL that DM causes is modest compared to those caused by such diseases. However, in order to interpret properly these figures, it should be taken into consideration the information provided in Table 2, as it indicates that most people who suffer from DM also have vascular risk factors and/or cardiovascular diseases. The combined effects of these elements make the loss of HRQoL associated with DM comparable to the three diseases mentioned previously.

In our study, HRQoL has also been analysed by dimension (mobility, self-care, daily activities, pain and discomfort, and anxiety/depression). The main differences between people with diabetes and the control group are shown in mobility, daily activities, and feeling pain or discomfort. More specifically, people with diabetes are 14.5 percentage points more likely to have mobility problems in comparison to those with neither diabetes nor any cardiovascular risk factors or diseases (control group). Furthermore, people with diabetes are 11.2 percentage points more likely to have difficulties with daily activities, and 15 percentage points more likely to feel pain or discomfort, in comparison to the control group. However, very few international studies have used large samples to compare different dimensions of HRQoL between people with and without diabetes [36, 37]. Notwithstanding, our results cannot be directly compared to those obtained from these studies, as they applied different instruments to measure HRQoL. Moreover, they only reported a descriptive analysis based on the percentages of people with and without diabetes in each HRQoL dimension, and did not show the differences for each dimension of HRQoL between people with and without diabetes, as we have done.

With regards to the limitations of the study, given the nature of the data, it has not been possible to perform a longitudinal analysis of individual health status over time, which would enable a deeper knowledge of changes in HRQoL when groups are diagnosed with diabetes or cardiovascular risk factors, or survive a cardiovascular event. On the other hand, the body mass index has been estimated based on the available data for weight and height given by the respondents, so answers can be sensitive to potential misreporting and recall bias or even to social norms [38, 39]. Additionally, this survey has the advantage of being representative of the general Spanish population, and includes extensive information on not only the sociodemographic characteristics, but also on the health status of the population and the utilization of healthcare resources; however, it is not a survey specifically designed to study health problems in people with diabetes. For this reason, the effect of specific diabetes complications with great impact in HRQOL as neuropathy, retinopathy, amputations or hypoglycaemia [27–32] could not be identified. Moreover, as there is a significant percentage of undiagnosed patients, some people who have denied having diabetes could actually have the disease [40].

## Conclusion

In brief, our findings suggest; (i) there are no significant differences on HRQoL of people with diabetes without cardiovascular risk factor or cardiovascular events vs people without diabetes without cardiovascular risk factor or cardiovascular events; (ii) when different cardiovascular risks and cardiovascular disease are present, differences between people with or without diabetes become statistically significant and negative; (iii) the cardiovascular risk which has the highest impact on HRQoL of people with diabetes is obesity; and (iv) cardiovascular disease has a stronger impact in the deterioration of HRQoL than any other cardiovascular risk factor, even when people with diabetes have the presence up to the three of them. Although the size of the effect estimated in this study may look small, the results are quite relevant from a clinical point of view, as it is necessary to consider that cardiovascular disease is the most frequent complication in people with diabetes, especially in older people with diabetes, representing more than 50% of people with diabetes. Moreover, diabetes is one of the leading causes of an increase in Disability-Adjusted Life Years (DALYs) recently reported by the Global Burden of Disease Study [41] and one of the potential explanations for this impairment comes from the effect of diabetes on cardiovascular diseases, which promotes disability and finally impact on QoL, Accordingly, our results could be of great value for the design, implementation, and evaluation of preventive policies and therapeutic programs focused on reducing the incidence and prevalence of cardiovascular risk factors, mainly obesity, and cardiovascular diseases, which reduce quality of life the most. The identification of programs and interventions that bring together a rational and efficient use of resources, with elements of distributive equity in terms of access and benefits, is compulsory for health authorities. In this study, we have tried to report information that quantifies the potential loss in quality of life that might be avoided with programmes brought to the population with diabetes. However, it remains to be seen whether programs aimed at improving glycaemic control could prevent or delay negative health shocks.

## Supporting information

S1 Table Main sociodemographic characteristics of the sample. (DOCX)

S2 Table Results from the matching regressions detailed by cardiovascular risk factor. HRQOL-TTO score.
(DOCX)

S3 Table Results from the matching methods applied. Dimension 1: Mobility.
(DOCX)

S4 Table Results from the matching methods applied. Dimension 2: Self-care.
(DOCX)

S5 Table Results from the matching methods applied. Dimension 3: Usual activities.
(DOCX)

S6 Table Results from the matching methods applied. Dimension 4: pain/discomfort.
(DOCX)

S7 Table Results from the matching methods applied. Dimension 5: anxiety and depression.
(DOCX)
