# Supplementary material for: Is quality of life different between diabetic and non-diabetic people? The importance of cardiovascular risks
Source: PLoS One. 2017 Dec 14;12(12):e0189505. doi: 10.1371/journal.pone.0189505 (PMC5730158; doi:10.1371/journal.pone.0189505)
Supplement: S1 Table — (DOCX) [file pone.0189505.s001.docx]

**S1 SUPPORTING INFORMATION**

Table s1. Main sociodemographic characteristics of the sample

|  | **People with DM** | **People without DM** | **People without DM (≥55)** |
| --- | --- | --- | --- |
| **Age (SD)** | 67.86 (13.54) | 49.91 (18.76) | 69.63 (9.98) |
| **Gender (%)** |  |  |  |
| Male | 45.35 | 45.97 | 40.01 |
| Female | 54.65 | 54.03 | 59.99 |
| **Marital Status (%)** |  |  |  |
| Single | 10.50 | 29.92 | 10.02 |
| Married | 54.54 | 52.06 | 56.47 |
| Widowed | 29.61 | 11.38 | 27.48 |
| Divorced | 5.30 | 6.56 | 5.97 |
| NA | 0.05 | 0.08 | 0.06 |
| **Education (%)** |  |  |  |
| No studies | 7.98 | 1.77 | 4.02 |
| Primary studies | 48.71 | 22.58 | 44.37 |
| Secondly studies | 37.84 | 59.76 | 42.99 |
| Higher studies | 5.47 | 15.89 | 8.62 |
| **Labour situation (%)** |  |  |  |
| Employed | 15.96 | 41.87 | 15.58 |
| Unemployed | 5.04 | 12.93 | 4.41 |
| Retire | 56.33 | 21.93 | 55.33 |
| Student | 0.21 | 6.37 | 0.01 |
| Disabled | 2.83 | 1.94 | 1.78 |
| Housework | 19.32 | 14.59 | 22.56 |
| Others | 0.31 | 0.37 | 0.32 |
| **Nationality** |  |  |  |
| Spanish | 98.11 | 93.25 | 97.79 |

Source: Authors’ version, based on the National Health Survey
