# Supplementary material for: Is quality of life different between diabetic and non-diabetic people? The importance of cardiovascular risks
Source: PLoS One. 2017 Dec 14;12(12):e0189505. doi: 10.1371/journal.pone.0189505 (PMC5730158; doi:10.1371/journal.pone.0189505)
Supplement: S2 Table — HRQOL-TTO score. (DOCX) [file pone.0189505.s002.docx]

**S2 SUPPORTING INFORMATION**

Table s2. Results from the matching regressions detailed by cardiovascular risk factor. HRQOL-TTO score.

| **Groups** | **Differences** | **S.D** | **T-stat** |
| --- | --- | --- | --- |
| People with diabetes & hypertension vs control group | -0.024 | 0.022 | -1.11 |
| People with diabetes & obesity vs control group | -0.042 | 0.022 | -1.86 |
| People with diabetes & hypercholesterolemia vs control group | -0.009 | 0.019 | -0.48 |
| People with diabetes & hypertension & hypercholesterolemia vs control group | -0.035 | 0.020 | -1.75 |
| People with diabetes & hypertension & obesity control group | -0.060 | 0.025 | -2.40 |
| People with diabetes & hypercholesterolemia & obesity vs control group | -0.005 | 0.038 | 0.14 |
|  |  |  |  |

Source: Authors’ version, based on the National Health Survey
