# Supplementary material for: Is quality of life different between diabetic and non-diabetic people? The importance of cardiovascular risks
Source: PLoS One. 2017 Dec 14;12(12):e0189505. doi: 10.1371/journal.pone.0189505 (PMC5730158; doi:10.1371/journal.pone.0189505)
Supplement: S3 Table — Dimension 1: Mobility. (DOCX) [file pone.0189505.s003.docx]

**S3 SUPPORTING INFORMATION**

Table s3. Results from the matching methods applied. Dimension 1: Mobility

|  | **Mobility** | | | | |
| --- | --- | --- | --- | --- | --- |
|  | **no problems** | **slight problems** | **moderate problems** | **severe problems** | **extreme problems** |
| **Group** | **Marginal effect (SD)** | **Marginal eff (SD)** | **Marginal eff(SD)** | **Marginal eff (SD)** | **Marginal eff (SD)** |
| People with diabetes vs control group | -0.1450  (0.015)* | 0.0174  (0.011) | 0.0648  (0.0106)* | 0.0495  (0.009)* | 0.0133  (0.004)* |
| People with diabetes without cardiovascular risk factor or cardiovascular event vs control group | -0.0088  (0.037) | 0.037  (0.025) | -0.028  (0.024) | 0.014  (0.018) | -0.0142  (0.008) |
| People with diabetes with cardiovascular risk factors and without cardiovascular event vs control group | -0.117  (0.0201)* | 0.030  (0.015)* | 0.048  (0.013)* | 0.028  (0.010)* | 0.011  (0.005)* |
| People with diabetes with cardiovascular event vs control group | -0.240  (0.029)* | 0.013  (0.024) | 0.095  (0.023)* | 0.097  (0.021)* | 0.037  (0.010)* |
| People without diabetes with cardiovascular risk factors and without cardiovascular events vs control group | -0.037  (0.006)* | 0.017  (0.005)* | 0.017  (0.004)* | 0.004  (0.002) | -0.001  (0.001) |
| People without diabetes with cardiovascular event vs control group | -0.193  (0.045)* | 0.031  (0.037) | 0.038  (0.034) | 0.094  (0.032)* | 0.030  (0.019)* |
| People with diabetes with 1 cardiovascular risk factor vs control group | -0.050  (0.030)* | 0.002  (0.021) | 0.020  (0.020) | 0.012  (0.016) | 0.016  (0.006)* |
| People with diabetes with 2 cardiovascular risk vs control group | -0.137  (0.031)* | 0.039  (0.023)* | 0.063  (0.022)* | 0.011  (0.017) | 0.024  (0.008)* |
| People with diabetes with 3 cardiovascular risk factors vs control group | -0.232  (0.051)* | 0.058  (0.042) | 0.093  (0.033)* | 0.058  (0.030)* | 0.023  (0.011)* |

*Statistically significant at 95% (p<0,05). Source: Authors’ version, based on the National Health Survey
