# Supplementary material for: Is quality of life different between diabetic and non-diabetic people? The importance of cardiovascular risks
Source: PLoS One. 2017 Dec 14;12(12):e0189505. doi: 10.1371/journal.pone.0189505 (PMC5730158; doi:10.1371/journal.pone.0189505)
Supplement: S4 Table — Dimension 2: Self-care. (DOCX) [file pone.0189505.s004.docx]

**S4 SUPPORTING INFORMATION**

Table s4. Results from the matching methods applied. Dimension 2: Self-care

|  | **SELF-CARE** | | | | |
| --- | --- | --- | --- | --- | --- |
|  | **no problems** | **slight problems** | **moderate problems** | **severe problems** | **extreme problems** |
| **Group** | **Marginal eff (SD)** | **Marginal eff (SD)** | **Marginal eff (SD)** | **Marginal eff (SD)** | **Marginal eff (SD)** |
| People with diabetes vs control group | -0.0658  (0.012)* | 0.014  (0.008)* | 0.0232  (0.007)* | 0.0216  (0.005)* | 0.007  (0.005) |
| People with diabetes without cardiovascular risk factors or cardiovascular event vs control group | 0  (0.027) | 0.014  (0.018) | -0.014  (0.014) | 0.028  (0.013)* | -0.028  (0.011)* |
| People with diabetes with cardiovascular risk factors and without cardiovascular event vs control group | -0.041  (0.015)* | 0.021  (0.010)* | 0.011  (0.009) | 0.010  (0.006)* | -0.001  (0.006) |
| People with diabetes with cardiovascular event vs control group | -0.140  (0.027)* | 0.037  (0.018)* | 0.035  (0.017)* | 0.031  (0.014)* | 0.037  (0.012)* |
| People without diabetes with cardiovascular risk factors and without cardiovascular disease vs control group | -0.007  (0.004) | 0.008  (0.003)* | 0.002  (0.002) | -0.001  (0.001) | -0.002  (0.001) |
| People without diabetes with cardiovascular disease vs control group | -0.141  (0.042)* | 0.065  (0.028)* | 0.013  (0.026) | 0.042  (0.021)* | 0.021  (0.023) |
| People with diabetes with 1 cardiovascular risk factor vs control group | -0.010  (0.023) | 0.010  (0.016) | -0.012  (0.015) | 0.004  (0.009) | 0.008  (0.007) |
| People with diabetes with 2 cardiovascular risk factors vs control group | -0.037  (0.024)* | 0.016  (0.016) | 0.006  (0.013) | 0.013  (0.011) | 0.002  (0.008) |
| People with diabetes with 3 cardiovascular risk factors vs control group | -0.127  (0.040)* | 0.041  (0.029) | 0.052  (0.024)* | 0.011  (0.018) | 0.023  (0.014) |

*Statistically significant at 95% (p<0,05). Source: Authors’ version, based on the National Health Survey
