# Supplementary material for: Is quality of life different between diabetic and non-diabetic people? The importance of cardiovascular risks
Source: PLoS One. 2017 Dec 14;12(12):e0189505. doi: 10.1371/journal.pone.0189505 (PMC5730158; doi:10.1371/journal.pone.0189505)
Supplement: S5 Table — Dimension 3: Usual activities. (DOCX) [file pone.0189505.s005.docx]

**S5 SUPPORTING INFORMATION**

Table s5. Results from the matching methods applied. Dimension 3: Usual activities

|  | **Usual activities** | | | | |
| --- | --- | --- | --- | --- | --- |
|  | **no problems** | **slight problems** | **moderate problems** | **severe problems** | **extreme problems** |
| **Group** | **Marginal eff (SD)** | **Marginal eff (SD)** | **Marginal eff (SD)** | **Marginal eff (SD)** | **Marginal eff (SD)** |
| People with diabetes vs control group | -0.1175  (0.014)* | 0.0406  (0.010)* | 0.0406  (0.009)* | 0.0121  (0.006)* | 0.0242  (0.006)* |
| People with diabetes without cardiovascular risk or cardiovascular disease vs control group | 0.023  (0.032) | -0.0047  (0.023) | -0.023  (0.0155) | 0.018  (0.014) | -0.014  (0.014) |
| People with diabetes with cardiovascular risk factors and without cardiovascular disease vs control group | -0.086  (0.018)* | 0.047  (0.013)* | 0.034  (0.011)* | -0.003  (0.007) | 0.008  (0.008) |
| People with diabetes with cardiovascular disease vs control group | -0.250  (0.029)* | 0.073  (0.022)* | 0.073  (0.020)* | 0.043  (0.016)* | 0.061  (0.016)* |
| People without diabetes with cardiovascular risk factors and without cardiovascular diseases vs control group | -0.021  (0.006)* | 0.019  (0.004)* | 0.004  (0.003) | -0.000  (0.002) | -0.002  (0.001) |
| People without diabetes with cardiovascular disease vs control group | -0.206  (0.045)* | 0.056  (0.034)* | 0.052  (0.031)* | 0.038  (0.022)* | 0.060  (0.028)* |
| People with diabetes with 1 cardiovascular risk vs control group | -0.036  (0.027) | 0.008  (0.020) | 0.028  (0.017) | -0.014  (0.010) | 0.014  (0.011) |
| People with diabetes with 2 cardiovascular risk vs control group | -0.085  (0.029)* | 0.042  (0.021)* | 0.031  (0.018)* | 0.006  (0.012) | 0.006  (0.012) |
| People with diabetes with 3 cardiovascular risk vs control group | -0.162  (0.048)* | 0.064  (0.038)* | 0.035  (0.029) | 0.034  (0.019)* | 0.029  (0.018) |

*Statistically significant at 95% (p<0,05). Source: Authors’ version, based on the National Health Survey
