# Supplementary material for: Is quality of life different between diabetic and non-diabetic people? The importance of cardiovascular risks
Source: PLoS One. 2017 Dec 14;12(12):e0189505. doi: 10.1371/journal.pone.0189505 (PMC5730158; doi:10.1371/journal.pone.0189505)
Supplement: S6 Table — Dimension 4: pain/discomfort. (DOCX) [file pone.0189505.s006.docx]

**S6 SUPPORTING INFORMATION**

Table s6. Results from the matching methods applied. Dimension 4: pain/discomfort

|  | **Pain/discomfort** | | | | |
| --- | --- | --- | --- | --- | --- |
|  | **no problems** | **slight problems** | **moderate problems** | **severe problems** | **extreme problems** |
| **Group** | **Marginal eff (SD)** | **Marginal eff (SD)** | **Marginal eff (SD)** | **Marginal eff(SD)** | **Marginal eff (SD)** |
| People with diabetes vs control group | -0.1500  (0.0160)* | 0.0416  (0.0131)* | 0.0685  (0.012)* | 0.032  (0.009)* | 0.0079  (0.003)* |
| People with diabetes without cardiovascular risk or cardiovascular disease vs control group | 0.061  (0.042) | -0.056  (0.032)* | 0.023  (0.026) | -0.033  (0.017)* | 0.005  (0.004) |
| People with diabetes with cardiovascular risk factors and without cardiovascular disease vs control group | -0.157  (0.020)* | 0.081  (0.016)* | 0.052  (0.015)* | 0.019  (0.011)* | 0.005  (0.002)* |
| People with diabetes with cardiovascular disease vs control group | -0.216  (0.029)* | 0.026  (0.025) | 0.090  (0.025)* | 0.076  (0.020)* | 0.024  (0.008)* |
| People without diabetes with cardiovascular risk factors and without cardiovascular diseases vs control group | -0.064  (0.008)* | 0.027  (0.006)* | 0.029  (0.005)* | 0.007  (0.003)* | 0.001  (0.001) |
| People without diabetes with cardiovascular disease vs control group | -0.205  (0.044)* | 0.017  (0.039) | 0.094  (0.38)* | 0.098  (0.028)* | -0.004  (0.012) |
| People with diabetes with 1 cardiovascular risk vs control group | -0.090  (0.031)* | 0.036  (0.024) | 0.071  (0.023)* | -0.010  (0.015) | 0.002  (0.004) |
| People with diabetes with 2 cardiovascular risk vs control group | -0.136  (0.032)* | 0.088  (0.027)* | 0.033  (0.024) | 0.017  (0.017) | -0.002  (0.005) |
| People with diabetes with 3 cardiovascular risk vs control group | -0.250  (0.052)* | 0.081  (0.046)* | 0.081  (0.039)* | 0.081  (0.035)* | 0.007  (0.005) |

*Statistically significant at 95% (p<0,05). Source: Authors’ version, based on the National Health Survey
