# Supplementary material for: Is quality of life different between diabetic and non-diabetic people? The importance of cardiovascular risks
Source: PLoS One. 2017 Dec 14;12(12):e0189505. doi: 10.1371/journal.pone.0189505 (PMC5730158; doi:10.1371/journal.pone.0189505)
Supplement: S7 Table — Dimension 5: anxiety and depression. (DOCX) [file pone.0189505.s007.docx]

**S7 SUPPORTING INFORMATION**

Table s7. Results from the matching methods applied. Dimension 5: anxiety and depression

|  | **Anxiety and depression** | | | | |
| --- | --- | --- | --- | --- | --- |
|  | **no problems** | **slight problems** | **moderate problems** | **severe problems** | **extreme problems** |
| **Group** | **Marginal eff (SD)** | **Marginal eff (SD)** | **Marginal eff (SD)** | **Marginal eff (SD)** | **Marginal eff (SD)** |
| People with diabetes vs control group | -0.080  (0.014)* | 0.0358  (0.0109)* | 0.0300  (0.008)* | 0.0121  (0.005)* | 0.0021  (0.002) |
| People with diabetes without cardiovascular risk or cardiovascular disease vs control group | 0.023  (0.033) | -0.014  (0.026) | -0.018  (0.017) | 0.014  (0.014) | -0.005  (0.004) |
| People with diabetes with cardiovascular risk factors and without cardiovascular disease vs control group | -0.068  (0.018)* | 0.043  (0.014)* | 0.021  (0.011)* | 0.004  (0.006) | -0.000  (0.003) |
| People with diabetes with cardiovascular disease vs control group | -0.167  (0.027)* | 0.058  (0.021)* | 0.067  (.018)* | 0.037  (0.012)* | 0.005  (0.006) |
| People without diabetes with cardiovascular risk factors and without cardiovascular diseases vs control group | -0.035  (0.006)* | 0.016  (0.005)* | 0.013  (0.003)* | 0.006  (0.002)* | 0.000  (0.001) |
| People without diabetes with cardiovascular disease vs control group | -0.132  (0.042)* | 0.051  (0.032)* | 0.038  (0.027) | 0.051  (0.021)* | -0.008  (0.008) |
| People with diabetes with 1 cardiovascular risk vs control group | 0.002  (0.026) | -0.010  (0.021) | 0.016  (0.016) | -0.006  (0.009) | -0.002  (0.004) |
| People with diabetes with 2 cardiovascular risk vs control group | -0.092  (0.028)* | 0.061  (0.022)* | 0.033  (0.017)* | 0.004  (0.011) | -0.006  (0.006) |
| People with diabetes with 3 cardiovascular risk vs control group | -0.134  (0.048)* | 0.075  (0.038)* | 0.069  (0.029)* | -0.005  (0.019) | -0.005  (0.012) |

*Statistically significant at 95% (p<0,05). Source: Authors’ version, based on the National Health Survey
